# Normalizing Telemonitoring in Nurse-Led Care Models for Complex Chronic Patient Populations: Case Study

**DOI:** 10.2196/36346

**Published:** 2022-04-28

**Authors:** Kayleigh Gordon, Katie N Dainty, Carolyn Steele Gray, Jane DeLacy, Amika Shah, Emily Seto

**Affiliations:** 1 Institute of Health Policy, Management and Evaluation University of Toronto Toronto, ON Canada; 2 Centre for Global eHealth Innovation, Techna Institute, University Health Network Toronto, ON Canada; 3 North York General Hospital North York, ON Canada; 4 Bridgepoint Collaboratory for Research and Innovation, Lunenfeld-Tanenbaum Research Institute, Sinai Health System Toronto, ON Canada; 5 William Osler Health System Brampton, ON Canada

**Keywords:** telemonitoring, TM, nurse practitioner, NP-led care, models of care, integrated care, disease care model, disease, nurse, nurse-led implementation, complex chronic conditions, CCC, clinical team, mobile phone

## Abstract

**Background:**

The implementation of telemonitoring (TM) has been successful in terms of the overall feasibility and adoption in single disease care models. However, a lack of available research focused on nurse-led implementations of TM that targets patients with multiple and complex chronic conditions (CCC) hinders the scale and spread to these patient populations. In particular, little is known about the clinical perspective on the implementation of TM for patients with CCC in outpatient care.

**Objective:**

This study aims to better understand the perspective of the clinical team (both frontline clinicians and those in administrative positions) on the implementation and normalization of TM for complex patients in a nurse-led clinic model.

**Methods:**

A pragmatic, 6-month implementation study was conducted to embed multicondition TM, including heart failure, hypertension, and diabetes, into an integrated nurse-led model of care. Throughout the study, clinical team members were observed, and a chart review was conducted of the care provided during this time. At the end of the study, clinical team members participated in qualitative interviews and completed the adapted Normalization Measure Development questionnaires. The Normalization Process Theory guided the deductive data analysis.

**Results:**

Overall, 9 team members participated in the study as part of a larger feasibility study of the TM program, of which 26 patients were enrolled. Team members had a shared understanding of the purpose and value of TM as an intervention embedded within their practice to meet the diverse needs of their patients with CCC. TM aligned well with existing chronic care practices in several ways, yet it changed the process of care delivery (ie, interactional workability subconstruct). Effective TM normalization in nurse-led care requires rethinking of clinical workflows to incorporate TM, relationship development between the clinicians and their patients, communication with the interdisciplinary team, and frequent clinical care oversight. This was captured well through the subconstructs of skill set workability, relational integration, and contextual integration of the Normalization Process Theory.

**Conclusions:**

Clinicians successfully adopted TM into their everyday practice such that some providers felt their role would be significantly and negatively affected without TM. This study demonstrated that smartphone-based TM systems complemented the routine and challenging clinical work caring for patients with CCC in an integrated nurse-led care model.

## Introduction

In Canada, the prevalence of complex chronic conditions (CCC) is increasing nationwide, affecting 12% to 52% of individuals [1-4]. Patients with CCC include both those with multimorbidity and those who also face clinical complexities and challenges such as clinical or psychosocial vulnerability [5]. Growing evidence suggests that multimorbidity and complexity are driven not only by the nature of aging but also by lower socioeconomic status and social marginalization [3,5,6]. Polypharmacy, increasing medication dosages, and high dosage frequency are also associated with high rates of adverse events, poor adherence, and high treatment burden on the patient [7-10], advancing routine care needs. Negative outcomes related to multimorbidity occur beyond merely a summed effect of single conditions as conditions interact with each other, mutually enhancing their negative effects, leading to new clinical issues and unanticipated care trajectories that do not align with existing care guidelines [11].

Case management has emerged as a strategy to better integrate the care for complex patients [12], involving one central contact between the patient and their providers [13,14]. However, the results of integrated care are mixed [8,11], in part because of often inconsistent clinical interactions and numerous providers who fulfill the most responsible clinician role. Nurse-led care models involving advanced nurse practitioners (NPs) are emerging as a more distilled iteration of case management. Such models have been found to be effective [15-18] in part because of a more person-centered approach, inherent in nursing epistemology. The nursing approach is holistic, considering the broader context of psychosocial and sociocultural influences [19]. In Ontario, NPs have the legal authority to diagnose, prescribe, and treat patients, enabling immediate decision-making and clinical action [20]. As NPs are generally salaried providers versus fee-for-service, they are able to spend more time with patients who require additional support, incorporating different strategies to facilitate comprehensive and complete care [21]. Without appropriate monitoring routines or technologies, additional clinical efforts may be limited in their potential effect.

Digital interventions such as telemonitoring (TM) aim to support chronic disease self-management through routine and timely data transmission, enabling clinicians to identify symptom exacerbations early and intervene [22,23]. Several studies and systematic reviews have shown that TM can improve clinical communication and coordination as well as support patient self-management [23,24]. Although the implementation of TM has been clinically successful in single conditions [25-30], some studies have shown mixed results in areas of all-cause mortality after 365 days and 30-day readmission rates [31,32]. Mixed results could be because of the challenges associated with implementing new technologies without a clear understanding of existing workflows or an appropriate care model. In addition, there is limited research on TM for patients with CCC [33-35], which focuses on clinical workflows and care.

Implementing new models of care at scale is challenging [36], particularly in the context of clinical teams already working at capacity [37]. Normalization Process Theory (NPT) was used as a guide to determine the mechanisms (ie, conceptual or tangible) that contributed to or inhibited the process of embedding TM for CCC in an integrated nurse-led care model. NPT is a theory of implementation that focuses on the *work* involved to embed new interventions in their social context, detailing the mechanisms of coherence, cognitive participation, collective action, and reflexive monitoring [38-41]. Because of the importance of nurses’ role in the delivery of care in this model, it was important to understand their perspectives on implementing and using multicondition TM systems over 6 months and identify any barriers or facilitators. The feasibility and patient adherence to TM in nurse-led care, as well as the characteristics of the patients who used the TM app in the model have already been published [42].

The objective of this study is to better understand the perspective of the clinical team during a 6-month implementation of TM for complex patients in a nurse-led clinic model. Our research question was as follows: “Can TM be successfully implemented in an integrated-nurse-led model within the context of everyday clinical practice for patients with CCC?”

The research subquestions included the following:

1. Does the intervention make sense to clinical staff?
2. Are team members willing to engage with the intervention?
3. How can TM be successfully embedded into the clinical workflow of a nurse-led care model?
4. What can be learned about the overall implementation to increase the spread of TM-enabled nurse-led models of integrated care for patients with CCC?

## Methods

### Study Setting

In 2018, an NP-led integrated model of care for complex patients was established at a large ambulatory facility in Southern Ontario. The NP-led team of colocated interdisciplinary clinicians included an undergraduate-prepared registered nurse (RN), a pharmacist, a social worker, a kinesiologist, and a dietician. Specialists were also available for referrals based on patient’s previous care connections and new needs. Patients were seen in the clinic as often as weekly for 6 to 18 months, typically after an acute exacerbation or hospitalization. Although specialist appointments and routine primary care visits were not halted during the study period, the NP-led care model was intended to be the central coordinator for care, aiming to achieve clinical stabilization and optimization before repatriation back to routine primary care. In 2019, the smartphone-based TM platform *Medly* was integrated into the NP-led CCC model, enabling patients with multimorbidities including heart failure (HF), hypertension (HTN), and/or diabetes mellitus (DM) to record physiological metrics including blood pressure, weight, heart rate, blood sugar, and symptoms [42]. Although this list is not inclusive of all conditions associated with multimorbidity, they are common conditions that can be used as a foundation to realize a broader conceptualization of the tool in the future. On the basis of frequent readings at home, the algorithm generated self-care messages for patients and alerts for clinicians. Using a web-based dashboard containing a list of readings, alerts, and trend graphs, clinicians used their clinical judgment to conduct remote assessments, titrate medications, and determine further treatment actions. Critical alerts were sent by email to the NP and RN, although the RN conducted most monitoring duties during the study.

### Ethics Approval

All research activities were conducted with ethics approval from the William Osler Office of Research Ethics (#18-0061), the University Health Network Research Ethics Board (#18-5667), and the University of Toronto Research Ethics Board (#37660).

### Participants Sampling and Recruitment

There were 9 clinicians who were either frontline providers or administrators directly involved (ie, using the system in patient care or overseeing the TM in existing care workflows) in the implementation of the TM project and were invited via email to participate in the evaluation. Individuals were eligible to participate if they (1) were currently involved in the delivery of TM in the integrated nurse-led care model and (2) could speak and read in English. To maintain anonymity, the participants were not specifically identified by role. However, the roles included NPs, an RN, a social worker, a dietician, a pharmacist, a kinesiologist, a clinical manager, and senior administrators. Written informed consent was obtained from all participants.

### Data Collection and Analysis

#### Overview

A multimethod single-case study using an interpretivist paradigm was carried out to collect study data including in-depth interviews, supplemented with observations and questionnaires. All data were collected by the research coordinator (KG), an RN with frontline experience caring for patients with CCC and knowledge of diverse clinical operations using an interpretivist paradigm.

#### Key Informant Interviews

Qualitative in-depth interviews were conducted upon completion of the 6-month implementation. A semistructured interview guide was developed based on the 4 overarching constructs outlined in the NPT (Table 1) [38,40,43] and the study objectives. Interviews were conducted in person or over the phone based on the participant’s schedule, lasting between 30 minutes and over an hour. The interviews were audiotaped and professionally transcribed verbatim. In all cases, the purpose of the interviews was stated elicit individuals’ experiences and opinions about the implementation of TM in the nurse-led chronic care model and were aware that the interviewer was not involved in providing care services.

**Table 1:** NPT^a^ constructs and sample interview questions.

NPT construct	Construct definition	Interview questions
Coherence	Sense-making and understanding the purpose of the potential of the intervention	“Can you tell me about your role in the clinic? Can you tell me about your normal routine when you come into work? How do you compare TM^b^ to the current practices delivering complex chronic care?”
Cognitive participation	Buy-in and decision to commit to the work of the intervention	“You have been part of the clinic, which is utilizing TM, what is your understanding of TM?”
Collective action	The work that team members do to engage with the intervention	“Tell me about your experience working with and using TM? Do you feel it offers my benefits that are valued by team members? Do providers agree on the intent and benefit of using TM? Can you describe if and how TM affected your interactions with the patients?”
Reflexive monitoring	Reflection and appraisal of the intervention	“In your opinion, has your delivery of the TM program changed over time? Do you feel TM has contributed to patient care and patient self-care in the CMC?^c^ Are there factors that facilitate or inhibit TM in this care model? How could the TM system be changed or improved? How sustainable do you think the TM activities are in the long-term?”

^a^NPT: Normalization Process Theory.

^b^TM: telemonitoring.

^c^CMC: Complex Medicine Clinic.

#### In Situ Observation

Participants were observed throughout the entire study period. The research coordinator was embedded within the team to collect data through observations and field notes, typically 2 out of 5 days a week during the 6-month study period. Observations were made during patient visits, hallway conversations, team huddles, and group meetings to capture clinical discussions and workflows. Field notes were documented during the observations. The goal of this fieldwork was to generate comprehensive notes to better understand the implementation of TM by the clinicians through observing workflows and operations.

#### Chart Review

A chart review was also performed to provide a clinical context for the complexity and acuity of the patient participants in this case. Clinical data included the number of diagnosed chronic conditions, number of medications per patient, and frequency of health encounters. Chart review metrics (ie, number of conditions, medications, and frequency of visits) were analyzed descriptively to understand the patient population and to inform observations, as well as interview questions.

### Normalization Measure Development Questionnaire

The adapted Normalization Measure Development (NoMAD) questionnaire was administered to all participants upon study completion to supplement our understanding of the implementation processes in the nurse-led care model [44]. The 23-item NoMAD questionnaire represents a measure that was used to better understand participants’ experiences with the implementation of TM in nurse-led care. Each question was adapted to the questionnaire to reflect the study’s specific interventions. The word “telemonitoring” replaced the word “intervention” within the questionnaire. An email was sent as a reminder to complete the questionnaire during the last month of the study. Data within the NoMAD questionnaire were analyzed descriptively using absolute frequency and mean.

### Analysis Approach

An interpretative description approach was used to guide the qualitative analysis [45,46]. Multiple coders worked together to review and code the transcripts inductively. A tabular matrix was created to define each construct and how it might relate to the project, which was then used to deductively analyze a detailed summary of the codes based on the 4 main constructs and subconstructs of NPT (Multimedia Appendix 1). For example, there was some discrepancy between several codes within the relational integration subconstruct and contextual integration subconstruct, which resulted in further refinement of the meaning in the NP-led chronic care context until consensus was reached. Following the analysis, key themes, emerging connections, and alternative explanations were discussed. Themes and subthemes were reviewed for feedback with 2 participants in person as a form of synthesized member checking [47]. As part of the iterative interpretative description approach, other data sources from different clinical team members (ie, qualitative interview data, observation data, and the NoMAD questionnaire) were qualitatively analyzed for patterns and themes to make sense of the important ideas to be conveyed and access their meaning in a new context [47]. Furthermore, 2 researchers (KG and AS) independently read and reread the interview and observation memo transcripts and then met to discuss the findings until consensus was reached. Our intent was to compare codes with emerging themes and identify, if any, additional themes, data divergences, or other contextual factors related to the implementation. Convergence of the qualitative and quantitative data was examined by reviewing the results related to each NPT construct to further our understanding of the mechanisms that facilitate or inhibit TM of CCC in integrated nurse-led models of care.

## Results

### Overview

In total, 9 clinical team members agreed to participate in the implementation. However, 1 participant went on maternity leave shortly after the study started and therefore did not complete the questionnaire or participate in an interview. Another participant declined to participate in the postevaluations as they were not involved with the TM system in their role. Clinical team characteristics are listed in Table 2. In total, 86% (6/7) of the participants completed the questionnaires, and 100% (7/7) participants were interviewed about their experience. In addition, 2 (29%) participants were interviewed twice to clarify their statements and verify their intended meaning.

Table 2 presents the descriptive characteristics of the team. All the respondents were women. Overall, participants were highly experienced in their professional job category with the majority having >10 years of experience at the study institution and in their professional job category. Using the NoMAD general questions about the intervention, 67% (4/6) participants rated TM as familiar. In terms of coherence, most respondents agreed that TM was of value in their work (5/6, 83%), and all indicated that they would support TM in the future (cognitive participation). Interestingly, respondents had mixed opinions that TM could be easily integrated into the existing work (4/6, 67%). Regarding reflexive monitoring, most agreed that their feedback could be used to improve TM in the future (5/6, 83%).

**Table 2:** Clinical team characteristics.

Study ID	Age range (years)	Duration at study institution (years)	Duration in professional job category (years)	Current job category
CTM002	51-60	>15	>15	Frontline
CTM003	41-50	6-10	11-15	Frontline
CTM005	41-50	>15	11-15	Frontline
CTM006	31-40	<1	1-2	Frontline
CTM007	51-60	>15	3-5	Management
CTM008	61-70	>15	6-10	Management
CTM009	51-60	10-15	>15	Management

The following results present the perspectives of key team members involved in the implementation of TM within the integrated nurse-led model of care according to the NPT (Multimedia Appendix 2).

### Coherence: Does TM Make Sense as Part of Nurse-Led Complex Chronic Care Delivery?

#### Introducing TM and How the Requirements to Implement TM Were Processed

The mechanism of coherence focused on introducing TM as an intervention to support complex care and understanding how TM workflows might affect patient care preferences, clinical workflows, and care delivery practices. Processing the steps involved in implementing TM as well as the potential benefits of addressing current care challenges within existing individual workflows in the nurse-led care model was the first step in *sense-making*. Participants described specific challenges in managing CCC before implementation, including relying on snapshot assessments during in-person visits, poor patient recall, and patient handwritten or device logs. Given their reliance on patient-reported histories, clinicians struggled to obtain consistent and often accurate health data between appointments:

[Patients] bring a log. We ask them to write it down and then they bring a lot to their appointments. Some patients are very good with that, some patients are not. It all varies from patient to patient. Some are very compliant, some are not.

CTM002

Overall, participants found TM easy to understand and were familiar with its broad purpose. Several clinicians had previously used a TM system based on health coaching, allowing them to compare the new TM platform to their previous experience (ie, differentiation subconstruct) and envision the potential steps involved in this implementation. Some staff members felt that their complex patients may be apprehensive about TM technology. Clinicians wondered if their patients would use it and what kinds of benefits might be realized:

I think because we’ve had a long experience implementing Telehomecare...there’s a lot of similarities, there are differences in the case of Medly. I think the basic monitoring of patients is the same, it’s what you do with the information, who it’s going to, and how you react to it. The kind of response it generates...I think this is interesting because we’re looking at multiple conditions.

CTM008

#### Envisioning the Workflow Changes Required for TM Varied by Role

Individual participants reported similar understanding in providing TM for CCC (ie, providing visibility to the ongoing health status of their complex patients at home regularly might improve care overall). This contributed to a communal sense-making of how TM would work in a unique clinical model:

The ability to access patients virtually without them having to come into the hospital setting to receive care from a healthcare provider.

CTM009

Although all participants agreed on the purpose of TM, they had different views on the technology, the workload, and how each might affect their role (ie, the individual specification subconstruct). For the pharmacist, preexisting routines involved calling the patients weekly to obtain readings. The pharmacist thought that using TM would eliminate this process, creating more work time for other clinical responsibilities. By differentiating the workflow according to individual clinical responsibilities, the meaning of each clinician’s role with regards to TM began to make sense:

I think because we’ve had a long experience implementing [TM systems]. While there’s a lot of similarities, there are differences in the case of [this TM system]. I think the basic monitoring of patients is the same, it’s what you do with the information, who it’s going to watch it, and how you react to it.

CTM007

Field observations further solidified strong support for TM in CCC from senior leadership and the NP in charge by discussing TM and evaluating TM workflows and individual workloads. One administrator was observed to describe their support for clinician engagement with TM and facilitation of the work of TM in nurse-led care. Clinicians seemed to internalize the collective effort based on how TM was perceived to address the current care gaps (ie, monitoring escalating acuity and providing consistent patient communication) faced in practice, thus making a collective decision to implement TM.

### Cognitive Participation: Do Clinicians Engage With TM in Nurse-Led Chronic Care Delivery?

#### TM Training Varied by Clinical Responsibilities

Engagement with TM relied on clinicians to invest time in the go-live effort as well as throughout its implementation (ie, initiation subconstruct). During the implementation, the team engaged in multiple training sessions tailored to their roles. The process of learning to use TM centered around the individual’s workflow that varied among clinicians. The NP spent time with the study coordinator learning to use the system, and the RN spent time shadowing at another site, observing not only their unique site-specific workflows but also the system in the context of different clinical responsibilities (ie, enrollment subconstruct). Having the bulk of TM responsibilities within the RN workflow was initially described as stressful because of perceived added workload:

The first month was stressful and then that, it eased out...

CTM002

Other participants felt that the training was “straightforward” comparing similarities between the current care practices and TM tasks. This motivated clinicians to incorporate TM into daily work (ie, initiation subconstructs):

It was similar because we do ask them to check weight...blood pressure daily...and check their sugar, especially the patients who are on insulin.

CTM006

#### Aligning Workflows Supported a Willingness to Engage With TM and Clinical Buy-in

Before implementation, an appointment would include an assessment of the patients’ history, conditions, medications, and recent physiological trends. Clinicians would rely on patient-reported information, in-person physiological measurements, and devices to evaluate the patient status. Having remote readings readily accessible on a web-based dashboard in part legitimized the decision to invest time to align TM practices in their workflow and envision the future workflow benefits (ie, legitimation subconstruct). Aligning TM-embedded workflows with TM responsibilities facilitated clinician engagement in practice:

I have to look at the readings before we come up with a plan, whatever concerns there are, which part of the day their readings are up high and is it related to their meals? Are they carb loading, etc.? So that all depends on the readings. If I don’t have that info, my assessment is incomplete and if my assessment is incomplete, I’m not able to make a complete, more comprehensive care plan for the patient and with the patient.

CTM005

In determining the overall fit of TM within the current workflow practices on a day-to-day basis, participants felt that TM would be a good fit, particularly because of more frequent patient monitoring (ie, activation subconstruct) without creating additional appointments.

### Collective Action: How Can Multicondition TM Be Successfully Embedded in Nurse-Led Chronic Care Delivery?

#### Alert Management Aligned Well With Several Clinical Roles

Team members described how physiological readings were monitored within the integrated nurse model (ie, interactional workability subconstruct). TM alerts aligned well with certain more traditional RN responsibilities, such as clinical triage. The RN was responsible for monitoring the dashboard (ie, skill set workability subconstruct) and triaged relevant readings to the NP or other clinicians as clinically necessary:

[Name] used to keep tab on all the patients, on the alerts also…if she thought a patient would benefit from my following up or my talking to the patient, she would let me know and I used to speak with the patient on the phone and not wait for them to come in the clinic. So that was really helpful process. We could immediately tackle if there was any issue and not wait for the patient to actually come in so find out something was going wrong.

CTM006

Although the primary responsibility of monitoring was held by the RN, it was apparent that other clinicians were using TM routinely in practice (eg, the pharmacist and dietician). By sharing patients on TM, the team suggested that the workload could be more easily divided among other members of the team, given the similar clinical scope of others’ responsibilities in this care model. Communication of alerts would occur through normal triage methods, hallway check-ins, or daily clinical huddles. Given the similar skill sets and clinical responsibilities, the pharmacist noted that having alert emails would also improve their workflow:

I don’t have the emails so I think that would be of benefit. But I think that was one of the things that I kind of said in terms of improving the workflow. Right now, that’s not my responsibility to log onto see their alerts, so I think it would be nice to have more inclusivity of the entire team that is following some of these patients.

CTM003

#### TM Facilitated Sharing of the Clinical Workload

Clinicians frequently reviewed the TM dashboard, discussing TM during in-person patient appointments and during clinical case-review sessions with the entire team. For example, the responsibilities associated with the RN’s TM work were successfully transferred to the NP as part of a normal clinical handover (ie, skill set workability subconstruct) when the nurse went on a holiday. When probed, how this transition occurred, they described the monitoring responsibility as part of all other clinical responsibilities (ie, contextual integration subconstruct):

If [nurse name] is not here, then I just take over monitoring [TM name] when she is gone.

CTM006

Participants felt that TM facilitated communication within the model and between individual clinicians as they could all review the clinical dashboard, discuss the acuity and severity of the alert, and discuss the most appropriate clinical response (ie, contextual integration subconstruct):

It was actually the nurse mostly, [Name]. She was keeping a check on all the patients regularly and if she noticed something to do with patient blood sugar or their weight, those things, she would let me know and then I would call the patient back and would be working on that. That’s how we used to do it.

CTM005

In the new care model, participants felt that patients were more clinically acute than originally anticipated in the care model, often requiring significant nursing interventions (ie, intravenous medications or urgent bloodwork) during office visits. The work associated with TM (follow-up, titrating medications, referrals, etc) in combination with often unexpected and acute clinical interventions, was such that on several occasions an additional RN was brought in to implement additional nursing interventions (intravenous medications, electrocardiogram, and hypoglycemia management).

#### Knowing the Patient, Their Conditions, and Clinical Context Facilitated Active TM

Knowledge of the individual patient, their clinical conditions, and their specific clinical context was considered essential for successfully embedding the intervention in the nurse-led team (ie, contextual integration subconstruct). In one case, a TM alert identified a previously stable patient who was quickly decompensating. On the basis of an alert, the patient was brought in for an assessment and subsequently underwent emergent stent insertion. In this case, the combination of fluctuating weight and symptom reporting resulted in clinical action by the nurse-led team, which the care team deemed to have potentially saved the patient’s life:

...I talked to [name] (the NP) and we brought him in today...We had to send him over...to the cath lab. I think he will be staying a couple days, so he won’t be taking readings for the next few days.

CTM002-memo log

In another case, a patient was alerting frequently. However, after reviewing the readings and current trends, the team was able to identify that the patient had not answered the symptom questions as intended:

In the beginning I was calling him every day but now I know...once he put them in, he continues to do that. So, I don't call him every day now even if I see that specific alert, unless like I see ok he's having chest pain. If there's no change in your condition, don't answer yes, but he still continues sometimes. So now I know (that with him).

CTM002

Integrating TM in the social context of care involved patient caregivers as well. Clinical staff reported that caregivers would call the team about a TM reading or alert, indicating that they were also involved in TM, such as monitoring trends at home. This additional opportunity for communication, which otherwise would not likely have occurred (ie, without TM), facilitated providers’ understanding of the clinical context and continued use of the TM system as a tool to conduct complex care.

### Reflexive Monitoring: What Can Be Learned of Overall Implementation to Increase the Spread of TM-Enabled Nurse-Led Models of Integrated Care for Patients With CCC?

#### TM Enhanced Comprehensive Care of Patients With CCC

The ability to track and trend multiple readings over time, particularly those in the past, was reported as clinically informative (ie, systematization and communal appraisal subconstructs), changing the overall clinical workflow of care delivery in this model:

Even up until December, I was reviewing back, their blood pressure [from] back in August...

CTM006

Although specific health outcomes were not evaluated, clinicians reflected on how patients seemed to improve over time, which indicated the feasibility of TM within a nurse-led model from the provider’s perspective. All clinicians felt that the ability to monitor their acute patients more frequently was important; however, monitoring patients after intravenous interventions or other in-office procedures was thought to be particularly supportive in mitigating unanticipated changes in health status (ie, communal appraisal subconstruct):

It’s definitely been positive, for sure reduced emergency visits, because the alerts have triggered that connection. It’s also influenced – some of the ways that they’re reacting to these very complex patients...They know now that they can probably avoid an admission, they get an alert, a patient’s put on some weight or starting to feel breathless...

CTM007

Clinicians described faster response times to TM than originally intended, suggesting that TM for CCC was successfully embedded as part of their normal work. This frequency of increased clinical oversight using TM contributed to sustained patient-provider communications:

I think [we responded] immediately. The moment we used to see any alert which needed attention, we used to do immediately. We never waited.

CTM005

At other times, alerts identified whether a patient had forgotten to take their readings or missed a medication, which otherwise may not have been noticed, indicating that TM enabled a higher level of care. In a few instances, participants found that patients depended heavily on the clinical team to reach out. For example, TM would prompt the patient to call the clinic if an alert was generated, but this did not happen routinely, in part because clinicians responded very quickly.

#### TM Provided a Reliable Routine Source of Health Data in Complex Chronic Care

TM provided reliable clinical data on patients, which were previously not available or consistent (ie, systematization subconstruct):

Some of our patients, they don’t record their blood pressure or they don’t bring their glucometers, it’s not easily known, so [with TM name] all the practitioners were able to access it and they did it without hesitation...sometimes they would forget to bring their readings or they don’t take their readings; now it’s all there, so it’s very convenient.

CTM005

Clinicians reported that TM provided additional contextual information before a patient’s appointment, which helped with the *prep* work of composing a clinical history and care plan. This enabled more clinically informed patients visits and made good use of the time allotted for structured appointments with each provider:

I think that allows me to do my prep work...I usually log on and get their trends, get all that information, so it’s a little bit more accurate, and I have time to sort of process it a little better than if they were to bring it in writing.

CTM003

Clinicians also described a better understanding of their patient’ health status when using TM in practice, demonstrating the relation integration of TM:

We’re able to follow them [the patients] more closely and if we get alerts that we would call them, it’s another sort of like a layer of protection in terms of if they’re running into trouble then we can follow or provide care.

CTM003

In terms of the overall patient population characteristics, associated clinical workload, and evaluation of how reliant (ie, embedded) TM had become in the clinic after 6 months, researchers asked what percentage of the other patients in their care might routine TM also be feasible. The overall response by clinicians was that they could envision most of their CCC patients benefiting from TM:

I think majority of our patients can be on [TM name]. It worked well for almost everyone we added.

CTM006

#### Reconfigurations Suggested for TM Implementations in NP-Led Complex Care Models

In this implementation, the TM platform monitored 3 conditions (HF, HTN, and DM). However, clinicians identified several other conditions as highly prevalent within their complex patients (ie, reconfiguration) but also in the broader chronic care population. Opportunities to monitor mental health symptoms and/or conditions were similarly identified across participants:

We have a lot of people with anxiety and even depression, it’s very prevalent in our patient population. You know we have the social worker for that very reason...Even if the Medly could have some questions about their mental well-being, I think that would help us.

CTM 006; CTM006-memo log

The inability to monitor respiratory status in patients with chronic obstructive pulmonary disease (COPD) was identified as an area that would be clinically helpful in addition to the existing TM platform. Other routine metrics such as temperature have also been identified as important to monitor in future TM implementations:

For COPD, the temperature would be really helpful. Especially now, we are going into the winter season, it would be really helpful to have temperature for those patients [when they get flu-like symptoms] maybe even those with CHF as well...

CTM006

Clinicians felt that visual differentiation of alerts by color (ie, critical vs noncritical) would be helpful in both the dashboard and email notifications, thereby designating a visual cue to quickly evaluate alert severity (ie, reconfiguration). The incorporation of TM into the routine care context in this model (ie, contextual integration) is such that a visual cue to identify critical needs could improve the efficiency of the monitoring process:

if we could get the system to identify the alert severity by color that would be very helpful...even if I only have just enough time to look at it quickly, I can see who is critical.

CTM002-memo log

## Discussion

### Principal Findings

This study explored the perceptions and experiences of clinicians regarding the implementation of a TM platform for complex patients as part of a larger feasibility study in an integrated nurse-led model. Our findings suggest that TM made sense to the colocated nurse-led team by considering individually and collectively how this work might affect their current care preferences, workflows, and existing delivery practices. Team members were willing to engage with the intervention as it aligned with tasks the team had already asked patients to do, such as monitoring blood pressure or weight at home regularly. A few clinicians had prior experience with the implementation of a TM system, which likely influenced their willingness to engage in and adopt the *Medly* system, which was evidenced by individuals comparing their experiences. Continuous patient engagement with the clinicians occurred frequently using TM and was considered fundamental in this case. Previous literature has indicated that regular interactions facilitated continued monitoring, further dialogue around disease management, documentation of new symptoms, and opportunities to identify health issues before they become critical [48,49].

TM aligned well with traditional RN responsibilities of assessment, communication, evaluation, triage, and delegation [50-52]. However, in this case, the RN had significant clinical responsibilities in addition to those of the TM, which may or may not be unique to other complex chronic disease management models. Previous research in TM of single conditions has suggested that nurses are well-positioned to manage TM requirements owing to the nature of the nurse-patient relationship [53], ability to analyze and apply TM data sources, multitasking, and providing a service that is fit for purpose [51,52,54]. However, other clinicians, particularly the pharmacist and dietician, routinely used TM data throughout the implementation in practice, suggesting that these roles might be better used in TM implementation in future. Previous literature has supported the greater involvement of pharmacists, given their growing scope of practice [55]. Although the scope of pharmacists varies widely across Canadian provinces, the support for an expanded scope in chronic disease populations has gained traction [56,57] given their in-depth knowledge of medication management, pharmacological interactions, as well as sign and symptom assessment and evaluation.

In terms of technology, several reconfigurations have been proposed to improve the existing platform. At the time of this study, a comprehensive mental health module was not available despite frequent links in the literature to conditions such as anxiety and depression in patients with CCC [50,58,59]. However, given our findings in support of this concept, we would strongly encourage a mental health component within the existing platform and other TM implementations targeting chronic care. Other conditions, such as COPD and chronic kidney disease, are important for monitoring in future TM implementations, as they are highly prevalent in the population.

During the study, the team continued to identify other patients who might have benefited from TM but were not eligible because of the preapproved participant numbers. This appears to suggest that TM was successfully embedded within routine practice in NP-led care, and that the full benefits of TM-enabled NP-led integrated care models have yet to be fully realized for patients, families, and health care systems. A multisite implementation with larger participant volumes is needed to evaluate the effectiveness of individual health outcomes and the organizational impact of this model of care delivery.

### Implementation Learnings

Using NPT to evaluate the feasibility of implementation provided a foundation that can be used to enhance future research implementations as nurse-led care models expand and virtual care solutions such as TM for patients with CCC become more prevalent. Our findings suggest that TM-enabled nurse-led care is feasible, as demonstrated by its successful implementation in an ambulatory chronic care model. We offer the following learnings for clinicians, administrators, researchers, and policy makers to consider in developing and spreading nurse-led models for patients with CCC.

1. TM of physiological metrics for HF, HTN, and DM could be used in clinical practice, as monitoring aligned well with existing complex care practices.
2. RNs may be able to manage TM, in addition to their clinical responsibilities, in a nurse-led complex care model with a colocated multidisciplinary team.
3. The role of pharmacists in monitoring patients through TM should be considered because their responsibilities align well with TM triage and assessment. They could delegate tasks to the more responsible provider, in this case the RN or the NP as required.
4. TM-enabled workflows in chronic care still require complex needs to be triaged and directed as clinically necessary to the most responsible care provider (ie, the NP), as this maintains the centrally coordinated approach inherent within nurse-led care models.
5. TM platforms for complex chronic patients could consider incorporating the following:
  Methods of monitoring COPD and chronic kidney disease as these conditions are prevalent in the population of patients with CCC.
  Monitoring mental health conditions, particularly for anxiety and depression, could support diverse complex care needs.
  Easy to read, visual alert systems, including the use of color-coding systems to facilitate quick clinical evaluations of acuity and severity.

### Strengths and Limitations

This study had several notable strengths. First, TM was embedded into an existing nurse-led care model and not into a pilot clinic, a purposed clinic model, or a clinical model, contributing to the feasibility of TM in a real clinical environment that is actionable and informative. Second, the use of NPT strengthened the research by characterizing the core process elements of embedding TM in the implementation process, enabling researchers to describe the *work* involved in implementing TM in the NP-led model and contributing to existing research in the chronic care space. Third, unlike other TM implementations, patients were onboarded during regular clinical hours, avoiding additional appointments for TM onboarding or more expensive at-home visits for equipment setup and removal. Finally, patients within the complex care model were at times more medically acute, requiring a higher care level than envisioned initially in this outpatient model. Given the diversity and complexity inherent in multiple interacting diagnoses, the ability to manage this level of complexity using TM highlights the feasibility of TM for CCC in a nurse-led environment.

There were also several limitations to this study. First, interview data were collected upon study completion, and therefore, it was at times difficult to analyze clear differences in how the work was conducted (ie, mechanisms of collective action) and evaluation of the work processes (ie, reflexive monitoring) despite frequent clinical observations. Second, this study was conducted at a single site, but NP-led or nurse-led models of care could differ significantly in the scope of the care model, clinical roles, or target populations, thus hindering generalizability. Third, RN in this clinic had over 10 years of nursing experience, which may or may not be the case in future implementations. Finally, the NP-led care model was located within a large, multisite hospital system with access to existing clinical resources onsite, including a diagnostic imaging suite, laboratory, and urgent care center, and therefore, likely has access to clinical resources. This may have influenced the clinical workflow and normalization of TM in this case because access to these resources may not be generalizable to other sites. Other specialists were also available to support timely service delivery and provide additional clinical support to the clinic that likely shaped in part the overall clinician outlook of embedding TM in everyday routine practices.

### Conclusions

A TM platform for complex chronic patients was successfully implemented within an NP-led integrated care model. From the perspective of clinicians and administrators, the process of normalization occurred because TM aligned well with existing complex care practices of frequent assessment and evaluation. Similar to other TM implementations, RN responsibilities within the TM system aligned well with the existing practices of clinical triage, assessment, and evaluation. However, our results demonstrate that the role of pharmacists and dieticians within the infrastructure of an ambulatory NP-led model may also align well with TM responsibilities. The feasibility of a new model of TM-enabled care for CCC was indicated in this study, which provides evidence that such models of care should be further investigated to determine their effectiveness in improving clinical management and better patient outcomes. Our research also provides theoretically informed lessons and recommendations that can be applied to future implementations and studies.

## Appendix

Multimedia Appendix 1 Matrix of the operationalization of constructs and subconstructs of the Normalization Process Theory.

Multimedia Appendix 2 Themes from clinical team members.

## Abbreviations

CCC: complex chronic conditions
COPD: chronic obstructive pulmonary disease
DM: diabetes mellitus
HF: heart failure
HTN: hypertension
NoMAD: Normalization Measure Development
NP: nurse practitioner
NPT: Normalization Process Theory
RN: registered nurse
TM: telemonitoring

## References

[1] Sakib MN, Shooshtari S, St John P, Menec V (2019). The prevalence of multimorbidity and associations with lifestyle factors among middle-aged Canadians: an analysis of Canadian longitudinal study on aging data. BMC Public Health.

[2] Feely A, Lix LM, Reimer K (2017). Estimating multimorbidity prevalence with the Canadian Chronic Disease Surveillance System. Health Promot Chronic Dis Prev Can.

[3] Roberts KC, Rao DP, Bennett TL, Loukine L, Jayaraman GC (2015). Prevalence and patterns of chronic disease multimorbidity and associated determinants in Canada. Health Promot Chronic Dis Prev Can.

[4] Pefoyo AJ, Bronskill SE, Gruneir A, Calzavara A, Thavorn K, Petrosyan Y, Maxwell CJ, Bai Y, Wodchis WP (2015). The increasing burden and complexity of multimorbidity. BMC Public Health.

[5] Violan C, Foguet-Boreu Q, Flores-Mateo G, Salisbury C, Blom J, Freitag M, Glynn L, Muth C, Valderas JM (2014). Prevalence, determinants and patterns of multimorbidity in primary care: a systematic review of observational studies. PLoS One.

[6] Moin JS, Moineddin R, Upshur RE (2018). Measuring the association between marginalization and multimorbidity in Ontario, Canada: a cross-sectional study. J Comorb.

[7] Gallacher K, May CR, Montori VM, Mair FS (2011). Understanding patients' experiences of treatment burden in chronic heart failure using normalization process theory. Ann Fam Med.

[8] Aoki T, Yamamoto Y, Ikenoue T, Onishi Y, Fukuhara S (2018). Multimorbidity patterns in relation to polypharmacy and dosage frequency: a nationwide, cross-sectional study in a Japanese population. Sci Rep.

[9] van Merode T, van de Ven K, van den Akker M (2018). Patients with multimorbidity and their treatment burden in different daily life domains: a qualitative study in primary care in the Netherlands and Belgium. J Comorb.

[10] Mannucci PM, Nobili A, REPOSI Investigators (2014). Multimorbidity and polypharmacy in the elderly: lessons from REPOSI. Intern Emerg Med.

[11] Vetrano DL, Calderón-Larrañaga A, Marengoni A, Onder G, Bauer JM, Cesari M, Ferrucci L, Fratiglioni L (2018). An international perspective on chronic multimorbidity: approaching the elephant in the room. J Gerontol A Biol Sci Med Sci.

[12] Tortajada S, Giménez-Campos MS, Villar-López J, Faubel-Cava R, Donat-Castelló L, Valdivieso-Martínez B, Soriano-Melchor E, Bahamontes-Mulió A, García-Gómez JM (2017). Case management for patients with complex multimorbidity: development and validation of a coordinated intervention between primary and hospital care. Int J Integr Care.

[13] Wozniak L, Soprovich A, Rees S, Al Sayah F, Majumdar SR, Johnson JA (2015). Contextualizing the effectiveness of a collaborative care model for primary care patients with diabetes and depression (Teamcare): a qualitative assessment using RE-AIM. Can J Diabetes.

[14] Kastner M, Hayden L, Wong G, Lai Y, Makarski J, Treister V, Chan J, Lee JH, Ivers NM, Holroyd-Leduc J, Straus SE (2019). Underlying mechanisms of complex interventions addressing the care of older adults with multimorbidity: a realist review. BMJ Open.

[15] Heale R, James S, Garceau ML (2016). A multiple-case study in nurse practitioner-led clinics: an exploration of the quality of care for patients with multimorbidity. Nurs Leadersh (Tor Ont).

[16] Li S, Roschkov S, Alkhodair A, O'Neill BJ, Chik CL, Tsuyuki RT, Gyenes GT (2017). The effect of nurse practitioner-led intervention in diabetes care for patients admitted to cardiology services. Can J Diabetes.

[17] Hansen KT, McDonald C, O'Hara S, Post L, Silcox S, Gutmanis IA (2017). A formative evaluation of a nurse practitioner-led interprofessional geriatric outpatient clinic. J Interprof Care.

[18] Housden L, Browne AJ, Wong ST, Dawes M (2017). Attending to power differentials: how NP-led group medical visits can influence the management of chronic conditions. Health Expect.

[19] Carryer J, Adams S (2017). Nurse practitioners as a solution to transformative and sustainable health services in primary health care: a qualitative exploratory study. Collegian.

[20] (2019). Practice standard: nurse practitioner. College of Nurses of Ontario.

[21] Heale R, James S, Wenghofer E, Garceau ML (2018). Nurse practitioner's perceptions of the impact of the nurse practitioner-led clinic model on the quality of care of complex patients. Prim Health Care Res Dev.

[22] Desai AS, Stevenson LW (2010). Connecting the circle from home to heart-failure disease management. N Engl J Med.

[23] Aminuddin HB, Jiao N, Jiang Y, Hong J, Wang W (2021). Effectiveness of smartphone-based self-management interventions on self-efficacy, self-care activities, health-related quality of life and clinical outcomes in patients with type 2 diabetes: a systematic review and meta-analysis. Int J Nurs Stud.

[24] Yi JY, Kim Y, Cho YM, Kim H (2018). Self-management of chronic conditions using mHealth interventions in Korea: a systematic review. Healthc Inform Res.

[25] Seto E, Leonard KJ, Cafazzo JA, Barnsley J, Masino C, Ross HJ (2012). Mobile phone-based telemonitoring for heart failure management: a randomized controlled trial. J Med Internet Res.

[26] Seto E, Leonard KJ, Masino C, Cafazzo JA, Barnsley J, Ross HJ (2010). Attitudes of heart failure patients and health care providers towards mobile phone-based remote monitoring. J Med Internet Res.

[27] Seto E, Leonard KJ, Cafazzo JA, Masino C, Barnsley J, Ross HJ (2011). Mobile phone-based remote patient monitoring improves heart failure management and outcomes: a randomized controlled trial. J Am Coll Cardiol.

[28] Ware P, Dorai M, Ross HJ, Cafazzo JA, Laporte A, Boodoo C, Seto E (2019). Patient adherence to a mobile phone-based heart failure telemonitoring program: a longitudinal mixed-methods study. JMIR Mhealth Uhealth.

[29] Yun JE, Park JE, Park HY, Lee HY, Park DA (2018). Comparative effectiveness of telemonitoring versus usual care for heart failure: a systematic review and meta-analysis. J Card Fail.

[30] Pekmezaris R, Tortez L, Williams M, Patel V, Makaryus A, Zeltser R, Sinvani L, Wolf-Klein G, Lester J, Sison C, Lesser M, Kozikowski A (2018). Home telemonitoring in heart failure: a systematic review and meta-analysis. Health Aff (Millwood).

[31] Ong MK, Romano PS, Edgington S, Aronow HU, Auerbach AD, Black JT, De Marco T, Escarce JJ, Evangelista LS, Hanna B, Ganiats TG, Greenberg BH, Greenfield S, Kaplan SH, Kimchi A, Liu H, Lombardo D, Mangione CM, Sadeghi B, Sadeghi B, Sarrafzadeh M, Tong K, Fonarow GC, Better Effectiveness After Transition–Heart Failure (BEAT-HF) Research Group (2016). Effectiveness of remote patient monitoring after discharge of hospitalized patients with heart failure: the better effectiveness after transition -- heart failure (BEAT-HF) randomized clinical trial. JAMA Intern Med.

[32] Koehler F, Koehler K, Deckwart O, Prescher S, Wegscheider K, Kirwan BA, Winkler S, Vettorazzi E, Bruch L, Oeff M, Zugck C, Doerr G, Naegele H, Störk S, Butter C, Sechtem U, Angermann C, Gola G, Prondzinsky R, Edelmann F, Spethmann S, Schellong SM, Schulze PC, Bauersachs J, Wellge B, Schoebel C, Tajsic M, Dreger H, Anker SD, Stangl K (2018). Efficacy of telemedical interventional management in patients with heart failure (TIM-HF2): a randomised, controlled, parallel-group, unmasked trial. Lancet.

[33] Martín-Lesende I, Recalde E, Viviane-Wunderling P, Pinar T, Borghesi F, Aguirre T, Recio M, Martínez ME, Asua J (2016). Mortality in a cohort of complex patients with chronic illnesses and multimorbidity: a descriptive longitudinal study. BMC Palliat Care.

[34] Melchiorre MG, Lamura G, Barbabella F, ICARE4EU Consortium (2018). eHealth for people with multimorbidity: results from the ICARE4EU project and insights from the "10 e's" by Gunther Eysenbach. PLoS One.

[35] Lessard L, Amyot D, Aswad O, Mouttham A (2020). Expanding the nature and scope of requirements for service systems through service-dominant logic: the case of a telemonitoring service. Requirements Eng.

[36] Scott Kruse C, Karem P, Shifflett K, Vegi L, Ravi K, Brooks M (2018). Evaluating barriers to adopting telemedicine worldwide: a systematic review. J Telemed Telecare.

[37] Hammersley V, Parker R, Paterson M, Hanley J, Pinnock H, Padfield P, Stoddart A, Park HG, Sheikh A, McKinstry B (2020). Telemonitoring at scale for hypertension in primary care: an implementation study. PLoS Med.

[38] May CR, Mair F, Finch T, MacFarlane A, Dowrick C, Treweek S, Rapley T, Ballini L, Ong BN, Rogers A, Murray E, Elwyn G, Légaré F, Gunn J, Montori VM (2009). Development of a theory of implementation and integration: Normalization Process Theory. Implement Sci.

[39] McEvoy R, Ballini L, Maltoni S, O'Donnell CA, Mair FS, Macfarlane A (2014). A qualitative systematic review of studies using the Normalization Process Theory to research implementation processes. Implement Sci.

[40] May C, Finch T (2009). Implementing, embedding, and integrating practices: an outline of Normalization Process Theory. Sociology.

[41] May C (2013). Towards a general theory of implementation. Implement Sci.

[42] Gordon K, Dainty KN, Steele Gray C, DeLacy J, Shah A, Resnick M, Seto E (2020). Experiences of complex patients with telemonitoring in a nurse-led model of care: multimethod feasibility study. JMIR Nurs.

[43] May C (2013). Agency and implementation: understanding the embedding of healthcare innovations in practice. Soc Sci Med.

[44] Rapley T, Girling M, Mair FS, Murray E, Treweek S, McColl E, Steen IN, May CR, Finch TL (2018). Improving the normalization of complex interventions: part 1 - development of the NoMAD instrument for assessing implementation work based on normalization process theory (NPT). BMC Med Res Methodol.

[45] Thorne S, Kirkham SR, MacDonald-Emes J (1997). Interpretive description: a noncategorical qualitative alternative for developing nursing knowledge. Res Nurs Health.

[46] Thorne S (2016). Interpretative description: qualitative research for applied practice. 2nd edition.

[47] Thorne S, Kirkham SR, O'Flynn-Magee K (2004). The analytic challenge in interpretive description. Int J Qual Methods.

[48] Garnett A, Ploeg J, Markle-Reid M, Strachan PH (2018). Self-management of multiple chronic conditions by community-dwelling older adults: a concept analysis. SAGE Open Nurs.

[49] Boult C, Wieland GD (2010). Comprehensive primary care for older patients with multiple chronic conditions: "Nobody rushes you through". JAMA.

[50] Gordon K, Steele Gray C, Dainty KN, DeLacy J, Ware P, Seto E (2020). Exploring an innovative care model and telemonitoring for the management of patients with complex chronic needs: qualitative description study. JMIR Nurs.

[51] Grisot M, Moltubakk Kempton A, Hagen L, Aanestad M (2019). Data-work for personalized care: examining nurses' practices in remote monitoring of chronic patients. Health Informatics J.

[52] Bagot K, Moloczij N, Arthurson L, Hair C, Hancock S, Bladin CF, Cadilhac DA (2020). Nurses' role in implementing and sustaining acute telemedicine: a mixed-methods, pre-post design using an extended technology acceptance model. J Nurs Scholarsh.

[53] Vest BM, Hall VM, Kahn LS, Heider AR, Maloney N, Singh R (2017). Nurse perspectives on the implementation of routine telemonitoring for high-risk diabetes patients in a primary care setting. Prim Health Care Res Dev.

[54] Brewster L, Mountain G, Wessels B, Kelly C, Hawley M (2014). Factors affecting front line staff acceptance of telehealth technologies: a mixed-method systematic review. J Adv Nurs.

[55] Mc Namara KP, Breken BD, Alzubaidi HT, Bell JS, Dunbar JA, Walker C, Hernan A (2017). Health professional perspectives on the management of multimorbidity and polypharmacy for older patients in Australia. Age Ageing.

[56] Mansell K, Edmunds K, Guirguis L (2017). Pharmacists' scope of practice: supports for Canadians with diabetes. Can J Diabetes.

[57] Donald M, King-Shier K, Tsuyuki RT, Al Hamarneh YN, Jones CA, Manns B, Tonelli M, Tink W, Scott-Douglas N, Hemmelgarn BR (2017). Patient, family physician and community pharmacist perspectives on expanded pharmacy scope of practice: a qualitative study. CMAJ Open.

[58] Gould C, O'Hara R, Goldstein MK, Beaudreau SA (2016). Multimorbidity is associated with anxiety in older adults in the Health and Retirement Study. Int J Geriatr Psychiatry.

[59] Read JR, Sharpe L, Modini M, Dear BF (2017). Multimorbidity and depression: a systematic review and meta-analysis. J Affect Disord.

