# Correction: Normalizing Telemonitoring in Nurse-Led Care Models for Complex Chronic Patient Populations: Case Study

**DOI:** 10.2196/53833

**Published:** 2023-10-31

**Authors:** Kayleigh Gordon, Katie N Dainty, Carolyn Steele Gray, Jane DeLacy, Amika Shah, Emily Seto

**Affiliations:** 1 Institute of Health Policy, Management and Evaluation University of Toronto Toronto, ON Canada; 2 Centre for Global eHealth Innovation, Techna Institute, University Health Network Toronto, ON Canada; 3 North York General Hospital North York, ON Canada; 4 Bridgepoint Collaboratory for Research and Innovation, Lunenfeld-Tanenbaum Research Institute, Sinai Health System Toronto, ON Canada; 5 William Osler Health System Brampton, ON Canada

In “Normalizing Telemonitoring in Nurse-Led Care Models for Complex Chronic Patient Populations” (JMIR Nursing 2022;5(1):e36346) the authors made two additions.

In the original publication, one author was missed and the existing authors were incorrectly arranged. The authorship list was originally published as:

*Kayleigh Gordon, Emily Seto, Katie N Dainty, Carolyn Steele Gray, Jane DeLacy*

This has been changed to read as follows:

*Kayleigh Gordon, Katie N Dainty, Carolyn Steele Gray, Jane DeLacy, Amika Shah, Emily Seto*

Additionally, an Acknowledgments section has been added as follows:

*This research was made possible by the funding support from a Canadian Institutes of Health Research Personalized Health Catalyst Grant (Funding Reference Number 155443).*

The correction will appear in the online version of the paper on the JMIR Publications website on November 1, 2023 together with the publication of this correction notice. Because this was made after submission to PubMed, PubMed Central, and other full-text repositories, the corrected article has also been resubmitted to those repositories.

