# Supplementary material for: Normalizing Telemonitoring in Nurse-Led Care Models for Complex Chronic Patient Populations: Case Study
Source: JMIR Nurs. 2022 Apr 28;5(1):e36346. doi: 10.2196/36346 (PMC9100369; doi:10.2196/36346)
Supplement: Multimedia Appendix 1 [file nursing_v5i1e36346_app1.docx]

Appendix 1: Matrix of the operationalization of constructs and subconstructs of NPT

| **Coherence**  **The idea and process change of TM** | **Cognitive Participation**  **Enrolment and engagement of team members** | **Collective Action**  **How the process of TM gets performed** | **Reflexive Monitoring**  **Appraisal of these new set of practices, and how it affects/changes them** |
| --- | --- | --- | --- |
| *Differentiation:* Understanding the difference between TM and traditional methods of face-to-face appointments.  *Communal specification:* Staff through teamwork create an understanding and process of routine monitoring at work.  *Individual specification:* Staff create an understanding of the new practice of TM.  *Internalised meaning:* The coherence of TM is agreed to and based on the meaning users collectively invest in it. | *Initiation*: Users’ motivation to incorporate TM into daily clinical work.  *Enrolment:* The work users do to organize themselves to utilize TM as part of their work.  *Legitimation:* Believing TM in the context of nurse-led care needs to complement the existing care plans and treatment plans  *Activation of TM:* The work users would need to do for TM to become a sustained part of the staff’s work daily life. | *Interactional workability:*  Monitoring readings at routine intervals.  *Relation integration:* Staff’s understanding of TM but also how and when to use it. Also understanding their colleagues’ expressions.  *Skill-set workability:* How monitoring responsibilities conducted and performed. This will influence how the work is defined divided among users.  *Contextual Integration:*  The incorporation of TM into social context of the nurse-led care model. | *Systematisation:* The evaluation regarding TM’s usefulness and effectiveness.  *Communal appraisal:* Communal evaluation of the outcomes and value of TM.  *Individual appraisal:* Individual evaluation of outcomes and value of TM.  *Reconfiguration*: Opportunities for modification from staff that aim to modify and enhance TM. |
