# Supplementary material for: Normalizing Telemonitoring in Nurse-Led Care Models for Complex Chronic Patient Populations: Case Study
Source: JMIR Nurs. 2022 Apr 28;5(1):e36346. doi: 10.2196/36346 (PMC9100369; doi:10.2196/36346)
Supplement: Multimedia Appendix 2 [file nursing_v5i1e36346_app2.docx]

**Multimedia appendix 2.** Themes from clinical team members.

| NPT^a^ construct and subconstruct | | | Theme | Supporting quotes |
| --- | --- | --- | --- | --- |
| **Coherence** | | | |  |
|  | | Differentiation and individual specification | - Introducing TM^b^ and how the requirements to implement TM were processed | “There’s a lot of similarities, in this other application but there are differences in the case of Medly. I think the basic monitoring of patients is going to be the same, it’s what you do with the information, who the information is going to, and how you react to it, coordinate it. And...the kind of response it generates.” [CTM008] |
|  | | Communal specification and internalized meaning | - Envisioning the workflow changes required for TM varied by role | “My role was really to review, as a leader, the TM, in terms of is it feasible for us to go forward with my boss. And then it is just being involved in the hearing of the information from clinicians and bringing the staff onboard in terms of you know, we’re going to be doing this and how are we going to go about doing it.” [CTM009] |
| **Cognitive participation** | | | |  |
|  | | Initiation and enrollment | - TM training varied by clinical responsibilities | “The first month was stressful and then that, it eased out…In the beginning I really had a hard time making them [management] understand. No, I need time for this. Because they didn’t think that I need extra time for alerts.” [CTM002] |
|  | | Legitimation and activation | - Aligning workflows supported a willingness to engage with TM and clinical buy-in | “The technology is similar to the way we work with patients and what we ask the patients to do.” [CTM005] |
| **Collective action** | | | |  |
|  | | Interactional workability | - Alert management aligned well with several clinical roles | - “A convenience of having Medly is that it was always available. And you can go as far back as you want. But if the patient brought us a log only for like two weeks, then that’s all you had, whereas Medly you can go as far back as when they started.” [CTM006] - “Yes. So, we were aware, and she would say “Oh have you looked at this?” and I said, “Yes, I am going to.” [CTM002] |
|  | | Skill set workability | - Alert management aligned well with several clinical roles - TM facilitated sharing of clinical workload | - “There’s always a fine balance of how many people you want involved because we are still a small team but I can appreciate that, you don’t want everybody to be involved because then it’s like ‘well who’s going to be responsible because everyone got the alert so nobody does anything.’ Yeah, it’s better to have an individual...I think if it becomes an onus on just one person to do this then I think the entire team could be responsible and we should split up the patient list.” [CTM003] - “Yes…We discussed it [TM], if it was a major concern, then we discussed it as a team, like kind of like a hallway consult…It was more if there was no concern, we didn’t really discuss it and we kind of just reviewed it as needed.” [CTM006] |
|  | | Contextual integration and relational integration | - TM facilitated sharing of clinical workload | “It was actually the nurse mostly, [name]. She was keeping a check on all the patients regularly and if she noticed something to do with patient blood sugar or their weight, those things, she would let me know and then I would call the patient back and would be working on that. That’s how we used to do it.” [CTM005] |
| **Reconfiguration** | | | |  |
|  | | Systematization | - Knowing the patient, their conditions, and clinical context facilitated active TM | “Medly alerted…I talked to [name], the NP^c^ and we brought him in today. He was having some nocturnal chest pain, relieved by nitro spray, but way still have some ‘squeezing’ pain so we did the ECG^d^ and diagnosed as unstable angina…We had to send him over to the cath lab. I think he will be staying a couple days, so he won’t be taking readings for the next few days.” |
